# The effect of induced polyploidy on phytochemistry, cellular organelles and the expression of genes involved in thymol and carvacrol biosynthetic pathway in thyme (*Thymus vulgaris*)

**DOI:** 10.3389/fpls.2023.1228844

**Published:** 2023-09-15

**Authors:** Valiollah Mohammadi, Samaneh Talebi, Masoumeh Ahmadnasab, Hossein Mollahassanzadeh

**Affiliations:** Agronomy and Plant Breeding Department, University College of Agriculture and Natural Resources, University of Tehran, Karaj, Iran

**Keywords:** *Thymus vulgaris*, induced polyploidy, gene expression, thymol, carvacrol

## Abstract

Induced polyploidy usually results in larger vegetative and reproductive plant organs. In order to study the effect of chromosome doubling on *Thymus vulgaris*, three levels of colchicine concentration including 0.1, 0.3 and 0.5% (w/v) were applied for 6, 12 and 24 hours on apical meristem of 2- and 4-leaf seedlings. Ploidy level was evaluated by flow cytometry and microscopic chromosome counting. Chemical composition of essential oils extracted by hydro-distillation was analyzed by gas Chromatography/mass spectrometry (GC/MS) and gas Chromatography (GC). The application of 0.3% colchicine at 4-leaf seedling for 6 hours resulted in the highest survival rate and the highest number of tetraploid plants. Cytogenetic and flow cytometry analyses confirmed the increase of chromosome number from 2n=2x=30 in diploids to 2n=4x=60 in induced tetraploids. Tetraploid plants had larger leaves, taller and thicker stems, dense branching, longer trichome, larger stomata, larger guard cells, and decreased number of stomata. The number of chloroplasts and mitochondria increased significantly in tetraploid plants by 1.66 and 1.63 times, respectively. The expression of CYP71D178, CYP71D180 and CYP71D181 increased in tetraploids by 3.27, 7.39 and 2.15 times, respectively, probably resulting in higher essential oil compounds, as tetraploids outyielded the diploid plants by 64.7% in essential oil, 40.9% in thymol and 18.6% in carvacrol content.

## Introduction

Garden thyme *(Thymus vulgaris* L.*)* is one of the most important medicinal and aromatic plants. Besides common culinary and seasoning use, the antibacterial, anti-spasmolytic, antitumor and antiviral properties have made it very useful for pharmaceuticals especially for cough remedy (Satyal et al., 2016; Salma et al., 2017; Salehi et al., 2018; Mondak et al., 2020; Nieto, 2020; Galovičová et al., 2021). Very few efforts, however, have been made towards genetic improvement of this plant.

Increasing the number of chromosomes which is called ploploidy is one of the tools for plants improvement. Polyploidy has played an important role in plant breeding and also plant evolution (Sleper and Poehlman, 2006; Iannicelli et al., 2020; Madani et al., 2021). Winkler (1916) coined the term “polyploidy” in a study of vegetative grafts in *Solanum*, when he discovered tetraploid plants regenerated from callus tissue on cut stems of diploid *S. nigrum*. Winge (1917) proposed that spontaneous chromosome doubling, resulting from accidental somatic doubling in mitosis or non-reduction in meiosis, could convert interspecific sterile hybrids into fertile types. Grant (1981) postulated that 47% of all flowering plants were of polyploidy origin and proposed that 58% of monocots and 43% of dicots were polyploid. As polyploidization has made a great contribution to the evolution of many species, plant breeders try to take advantage of induced polyploidy in their programs. The hypothesis is that polyploidy may lead to useful characteristics like larger vegetative and reproductive organs (Adaniya and Shirai, 2001; Liqin et al., 2019; Madani et al., 2021). In addition, more than 90% of the reviewed experimental data point toward a positive impact of ploidy level on the ability of plants to cope with different kinds of biotic and abiotic stresses (Tossi et al., 2022).

Polyploidization can be induced by quite a few antimitotic agents. The most frequently used antimitotic chemicals are colchicine, trifluralin, and oryzalin. The whole method of induced chromosome doubling consists of a series of steps, including an induction phase, regrowth phase, and a confirmation technique to evaluate the rate of achievement (Salma et al., 2017). Blakeslee and Avery (1937) were among pioneers discovered the induction of polyploidy by colchicine. Induced polyploidy has been tried in several crop plants. Chen et al. (1945) generated autotetraploid barley plants by the use of colchicine and observed various irregularities in chromosome configuration as well as regular bivalents in a few lines. They also reported that the fertility of the 4n plants as a whole was lower than that of the diploids.

Compared with the original diploid, autotetraploid rice showed superior agronomic characters, such as larger panicle, larger seed and stronger stem, while seed-setting rate and fertility was lower than that in its diploid varieties (Song and Zhang, 1992; Chen et al., 2021).

Guo and Birchler (1994) studied dosage effects on gene expression in monoploid, diploid, triploid, and tetraploid maize plants and showed that for most tested genes, the transcript level per cell was directly proportional to structural gene dosage. Study of phenotypic and proteome changes in 2X, 4X and 6X ploidy series of maize revealed that plants of higher ploidy exhibited increased cell size but slower growth rate, later flowering, fewer tassel branches as well as reduced stature and fertility while the expression of most proteins increased linearly with ploidy (Yao et al., 2011).

Javadian et al. (2017) reported that induced tetraploidy increased podophyllotoxin content in the leaves and stems of *Linum album* by upregulating the expression level and enzyme activity of genes involved in the pathway.

Sabzehzari et al. (2019) demonstrated that induced tetraploid plants of Ispaghul (*Plantago psyllium*) were larger than their intact diploids in terms of height, leaf thickness, seed size, pollen grain, seeds per spike, number of guard cells and stomata size as well as chlorophyll and carotenoid content.

Besides experimental studies, induced polyploidy has long been used by plant breeders in commercial genetic improvement of sugar beet, seedless watermelon, rye, red clovers and ryegrass (Sattler et al., 2016). Polyploidy induction has also been widely applied to ornamental crops breeding, resulting in the extension of flower longevity, larger flower size and deep flower colors. Tulips and azalea are two examples with large number of induced polyploid commercial cultivars (Jones et al., 2007; Marasek-Ciolakowska et al., 2012)

Due to increased number of gene copies, artificially induced polyploidy may increase enzymatic content and activity that contribute to enhanced secondary metabolites of medicinal plants (Dhawan and Lavania, 1996; Iannicelli et al., 2020). The tetraploid forms of Chamomile (*Matricaria chamomilla* L) outperform their diploid relatives in both biochemical and morphological traits including essential oils, bisaboloids, chamazulene, apigenin, favonoids, height, capitulum size and weight and dry seed weight. Currently almost one quarter of the chamomile varieties cultivated worldwide are tetraploids induced by colchicine, highlighting the success of polyploidy breeding establishment in this medicinal crop (Das, 2014). The cultivated tetraploid chamomile varieties, Lutea and Goral, for instance, have been shown to produce 20% higher chamazulene (one of the valuable medicinal components of chamomile essential oil) than diploid cultivars (Gosztola et al., 2006). Tavan et al. (2015) used colchicine for ploidy induction in *Thymus persicus* nodal shoot tip explants and observed a higher amount of pentacyclic triterpenoids in induced tetraploids. Braynen et al. (2021) showed that the key genes promoting floral formation were down-regulated, whereas floral transition genes and a flowering locus were up-regulated in autotetraploid *Brassica rapa.* Iannicelli et al. (2020) reviewed the application of polyploidy in breeding of aromatic and medicinal species. Dhawan and Lavania (1996); Salma et al. (2017) and Madani et al. (2021) also reviewed the influence of artificial polyploidization on secondary metabolites content in medicinal plants.

Alsahlany et al. (2019) and Kramer and Bamberg (2019) studied guard cell size and number of chloroplasts per guard cell in potato trying to find a reliable simple tool to identify ploidy level. There has been very few attempts in the literature to induce polyploidy in *Thymus vulgaris*. Shmeit et al. (2020) treated nodal segments of thyme with 80μM centration of oryzalin for 24 hours and regenerated tetraploid plants on MS culture medium. They reported increased plant size and secondary metabolites in tetraploids. Navrátilová et al. (2021) tried *in vitro* polyploidization of diploid thyme explants by 0.346 and 1.73 mg L−1 oryzalin and developed tetraploid plants containing higher proportions of thymol and carvacrol. As both researches were carried out under resource- and facility-demanding *in vitro* condition, we aimed at this study to develop a more efficient colchicine-based protocol for *in vivo* polyploidization of *Thymus vulgaris* and to evaluate the its effect on physiological, anatomical, and phytochemical features.

## Material and methods

### Polyploidy induction

*Thymus vulgaris* seeds were planted in small pots filled with cocopeat and kept in a normal glass house with 16 hours day length, 26/18°C (day/night). Plants at 2- and 4-leaf stages were selected for colchicine treatment. Stock colchicine (Sigma^®^) solutions were prepared in sterilized distilled water. Two drops of 2% (v/v) dimethyl sulfoxide (DMSO) and Tween20 were added to increase cell permeability and colchicine absorption (Głowacka et al., 2009). 5 mL colchicine solution was dropped on apical meristem of seedlings and secured by a piece of cotton covered with aluminum foil to avoid evaporation. Three levels of colchicine concentration including 0.1, 0.3 and 0.5% (w/v) were applied for 6, 12 and 24 hours. Each treatment was run in 5 replications. Seedlings were then rinsed smoothly with water spray. Two weeks after treatment, plants were moved to bigger pots filled with soil, sand and cocopeat (3:2:1).

### Flow cytometry

Three months after seedlings treatment, plants showing atypical morphology (*i.e.* leaf thickness, shape, color, and size; shorter internodes) were selected as putative tetraploids. Ploidy level of selected plants were analyzed according to the method described by Loureiro et al. (2007) and Javadian et al. (2017). Soybean (*Glycine max*) variety Polanka (2C DNA = 2.50 pg) was used as standard reference plant (Dolezel et al., 1994). 25 mg leaf samples from putative plants as well as 25 mg from standard soybean were submerged in one mL Woody Buffer Plant (Loureiro et al., 2007) and chopped with a sharp razor blade. The suspension of isolated nuclei was filtered through a Partec 30 μm nylon mesh filters (Partec, Munster, Germany) to remove cell debris. The nuclear suspension of each sample was treated with 50 μg mL^-1^ RNase (Sigma-Aldrich Corporation, MO, USA) to prevent staining of double-stranded RNA and stained with μg mL^-1^ propidium iodide (PI, Fluka). Samples were then incubated on ice and analyzed by BDFACanto ™ flow cytometer (BD Biosciences, Bedford, MA, USA), using the BD FACSDiva™ Software. More than 5000 stained nuclei were analyzed per sample. Data were first transferred to Flowing Software version 2.5.0, followed by Partec FloMax Software version 2.4e (Partec, Munster, Germany). Instable plants with both diploid and tetraploid cells (mixoploids) were removed.

### Cytogenetic test

To confirm tetraploidy, chromosomes of the plants demonstrating DNA doubling in flow cytometry as well as control diploid plants were counted. 5-6 cm cuttings were made from each plant and regenerated to root. 1 cm long root tips were sampled and pretreated with 1% α-monobromonaphthalene for 3h at 4°C temperature followed by rinsing with distilled water for 10 min. Chromosome fixation was carried out using Carnoy’s solution consisting of 1part glacial acetic acid and 3 parts ethanol (95 to 100%) for 20 min at 4°C followed by 10 min rinsing with distilled water. Roots, then, hydrolyzed in 1 N HCl for 15 min at 60°C followed by 30 min rinsing. Specimens were stained with aceto orcein (2%) solution for 4 h. The stained root tips, 2 mm long, were squashed on a glass slide. The number of chromosomes was counted under Olympus DP70 light microscope equipped with a camera. Survival rate and tetraploid induction rate was calculated by the number of achieved tetraploid plants from each treatment divided by all treated plants.

### Measurement of morphological traits

All diploid and tetraploid plants were carefully monitored for important morphological traits after colchicine treatment. Plant height, leaf length, width and thickness, main stem diameter and internode distance were measured four months after treatment when the plants has become stable.

### Cellular features

**Figure 1:**
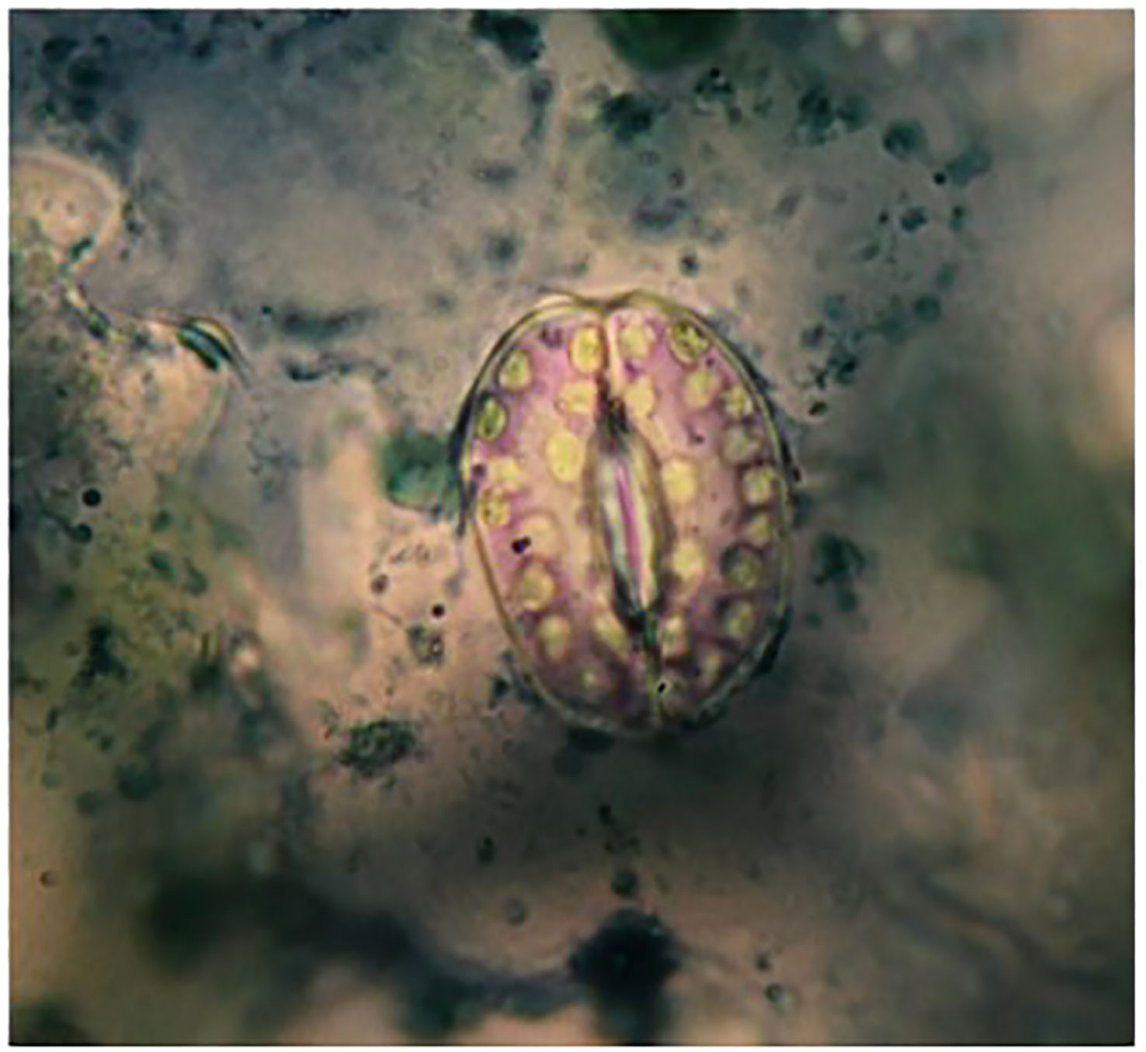
Guard cells from abaxial leaf epidermis of thyme plant stained.

Six diploid and six tetraploid plants (derived from 0.3% colchicine treatment for 6 hours at 4-leaf seedling) were selected for further cellular, physiological and biochemical measurements. Stomata length, width and density, trichome length and intensity as well as guard cells length and number (in a microscopic view scope) were measured using epidermis layer of three expanded leaves by colorless varnish (Smith et al., 1989). To make the epidermis layer prepared, the expanded leaves were dried, and the epidermis was separated using a piece of banderol. Separated epidermis were fixed on glass slides. The number of chloroplasts and mitochondria were counted in 150-200 guard cells in diploid and tetraploid plants’ leaves (**Figure 1**). 1% Janus Green powder (Merck™) solution was used to stain mitochondria.

### Phytochemical measurements

Thyme samples were dried at room condition (20–25° C) under shade for 72 hours. To assess the essential oil content, the shade dried samples were subjected to the hydro-distillation method for 3 hours with 250 mL water, using a Clevenger-type apparatus according to the British Pharmacopoeia (HMSO, 1988). Essential oil content was calculated by dividing mass essential oil to mass dry matter. The isolated oils were dried over anhydrous sodium sulfate and stored in tightly closed dark vials at 4°C for further analyses.

Gas chromatography (GC) analysis was performed using a Thermoquest gas chromatograph equipped with fused silica capillary DB-5 column (25 m×0.25 mm i.d.; film thickness 0.25 µm). The injector and detector temperatures were kept at 250 and 280°C, respectively. Nitrogen was used as the carrier gas at a flow rate of 1.1 mL/min; oven temperature program was 60–250°C at the rate of 4°C/min and finally held isothermally for 10 min; split ratio was 1:50.

Gas chromatography-mass spectrometer (GC-MS) analysis was carried out by use of Thermoquest-Finnigan gas chromatograph equipped with fused silica capillary DB-5 column (60 m×0.25 mm i.d.; film thickness 0.25 µm) coupled with a TRACE mass (Manchester, UK). Helium was used as carrier gas with ionization voltage of 70 eV. Ion source and interface temperatures were 200 and 250°C, respectively. Mass range was from 35 to 456 amu. Oven temperature program was the same as mentioned above for the GC. The constituents of the essential oils were calculated according to Hadian et al. (2014).

Total Chlorophyll (a+b) content was evaluated in tetraploid and diploid plants by the method described by Lichtenthaler (1987) using spectrophotometer (UV/Vis 2100 model). The absorbance was read by spectrophotometer (UV/Vis 2100 model) at 645 and 663 nm wavelengths. The pigments content was calculated based on the equations elucidated in Lichtenthaler and Wellburn (1983) and expressed as mg/g fresh weight.

### Gene expression

Total RNA was isolated and purified from thyme leaves using RNeasy plant mini kit (Qiagen, Valencia, CA, USA). The quality of the RNA samples was assessed in an agarose gel 2%, and its concentration was determined with a nanodrop. To remove contaminating DNA, RNAs were treated with DNaseI, RNase-free (Thermo. Fisher Scientific, Fermentas). DNase-treated RNA samples (1 μg) were reverse-transcribed using RevertAid™ First Strand cDNA Synthesis kit (Fermentas, K1622) to finally obtain a 20 μg cDNA. Specific primers for three cytochromes involved in thymol and carvacrol Biosynthesis including CYP71D178, CYP71D180 and CYP71D181 were designed based on Crocoll et al. (2010) and were checked by Primer3 Software with the following parameters: melting temperature of 60 °C, PCR product length range 120–200 bp pairs and Guanine-Cytosine contents from 50 to 60%. Real-time PCR (RT-PCR) was performed using the fluorescent SYBRGreen dye with BIOPRAD System. Relative quantification of gene expression was calculated by the 2^-ΔΔC^T method (Livak and Schmittgen, 2001) considering *Brassica napus* actin gene as the internal standard

### Data analysis

The impact of different concentration and duration of colchicine treatments at 2- and 4-leaf growth stages on survival rate and the number of tetraploid plants were statistically analyzed on the basis of completely randomized design ANOVA. The mean values were compared using the Duncan’s test by SAS 9 software (SAS Institute Inc. 2013).

## Results

### Polyploidy induction via colchicine treatment

**Figure 2:**
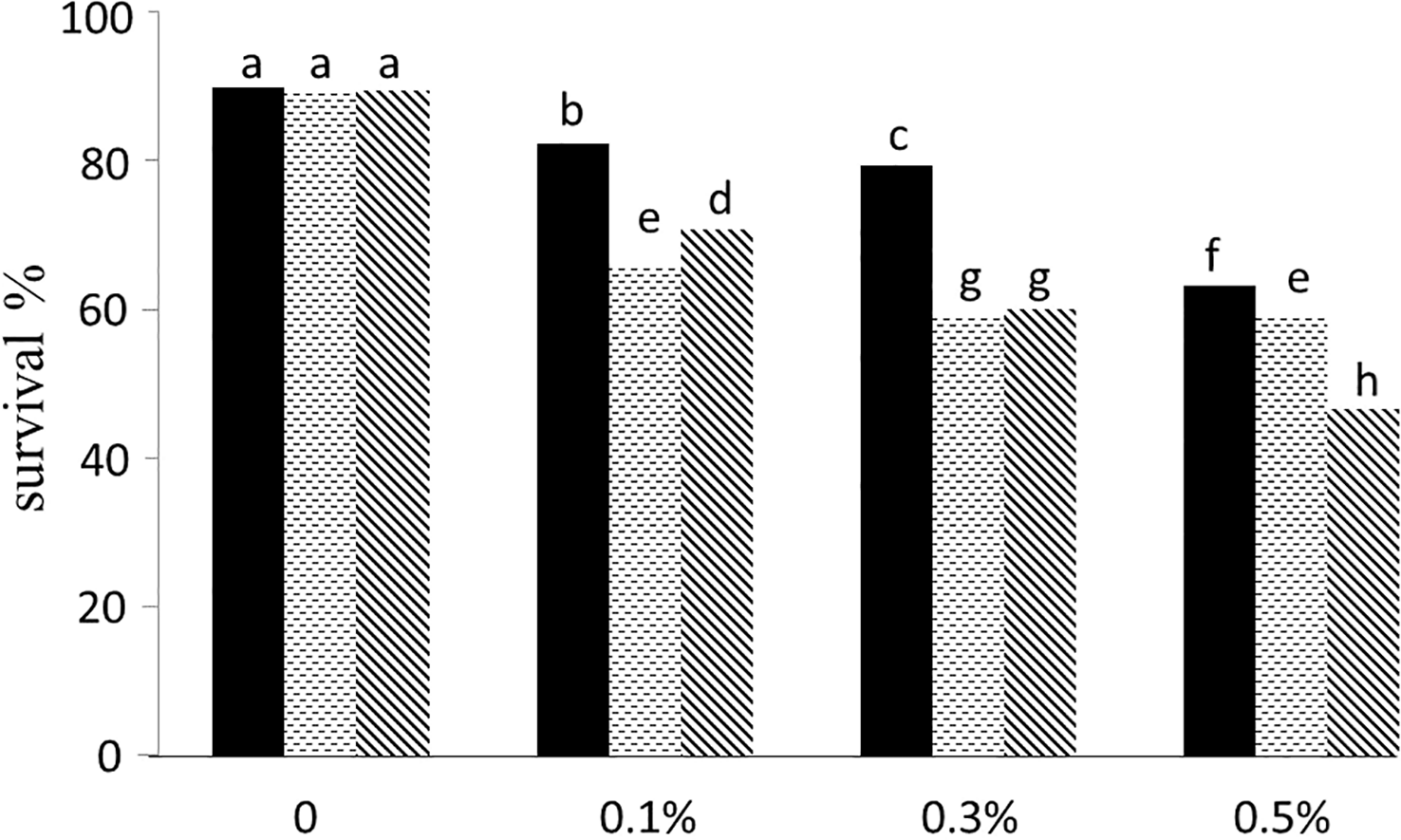
The impact of treatment time: 6 h (black bars), 12 h (dotted bars) and 24 h (hatched bars) and colchicine concentration on plants survival. The treatments with the same letter on top have no significant difference based on Duncan’s test in 0.01 significant level.

**Figure 3:**
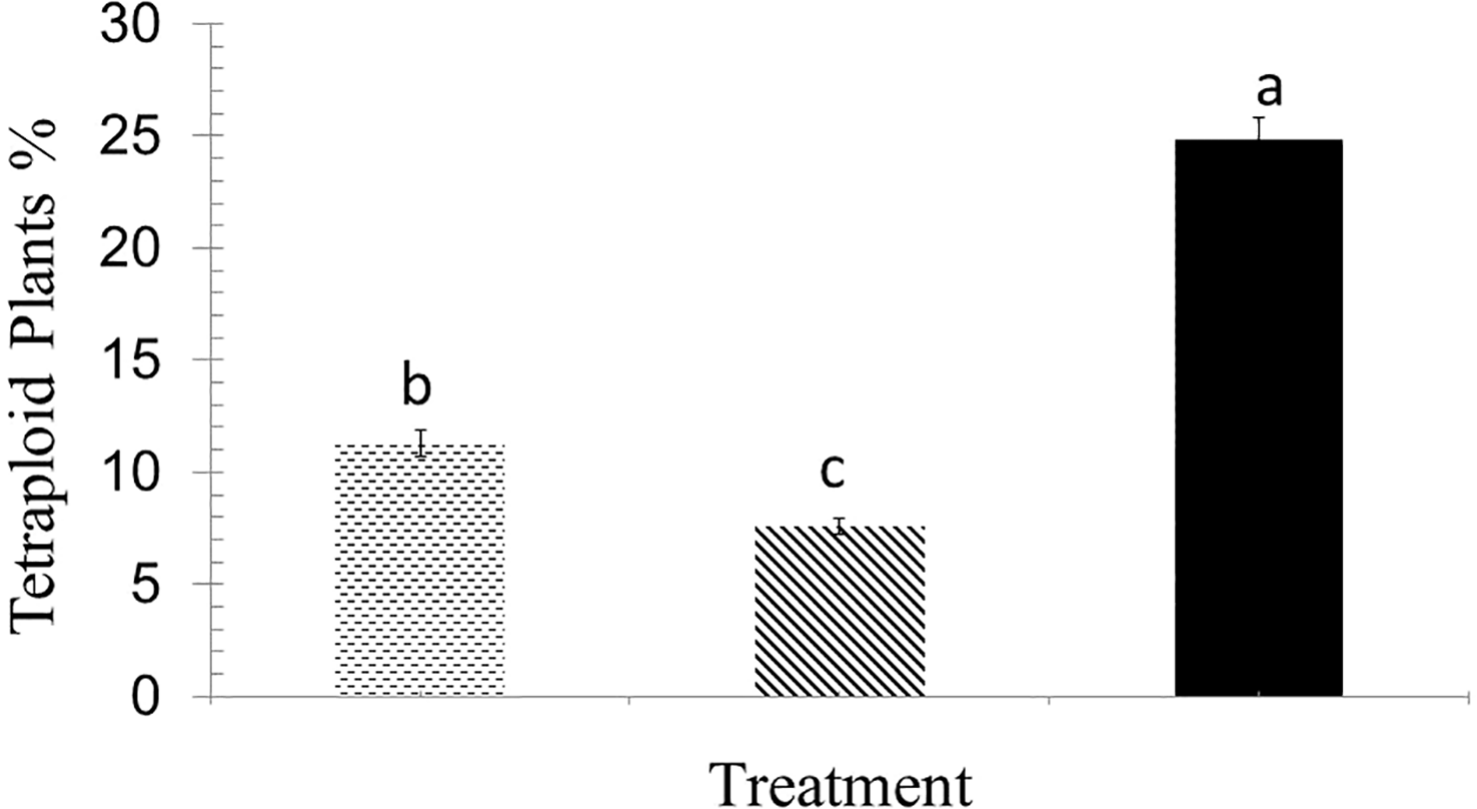
Percentage of tetraploid plants obtained from different treatments: 0.3% colchicine for 6 hours at 2-leaf stage (dotted bars), 0.1% colchicine for 6 hours at 4-leaf stage (hatched bars), and 0.3% colchicine for 6 hours at 4-leaf stage (black bars). The treatments with different letter on top are significantly different based on Duncan’s test by 0.01 significant level.

The higher concentration of colchicine resulted in less plant survival at both 2-leaf and 4-leaf stages (**Figure 2**). Out of 18 treatments including three concentrations of colchicine (0.1, 0.3 and 0.5%), three times of application (6, 12 and 24 hours), and two stages (2- and 4-leaf), six treatments failed to raise any polyploid plants. Only application for 6 hours of 0.3% colchicine at 2- and 4-leaf stage and 0.1% at 4-leaf stage appeared to be successful for inducing polyploidization, the very first being the most efficient one (**Figure 3**).

### Cytometry and cytogenetic test

**Figure 4:**
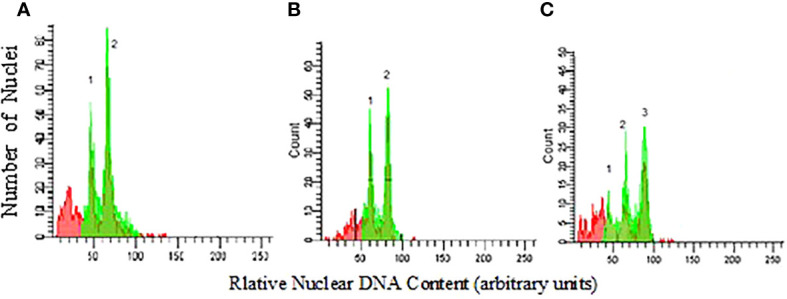
Flow cytometry analysis of thyme cell nuclei, **(A)** peak1 and 2: diploid and standard. **(B)** peak1 and 2: standard and tetraploid plant) **(C)** peak1-3: diploid, standard and tetraploid plants, respectively in a mixoploid. Soybean Polanka variety (2C DNA = 2.50 pg) was used as standard. Peaks C.V. kept less than 5%.

Plants showing morphological changes *e.g.*, darker leaf color, growth stunt, and low internode distance were chosen as candidate tetraploid plants and their ploidy level checked by flow cytometry and chromosome counting. The mean 2C DNA content of diploid thyme was found to be 1.88 picograms (pg) being very close to 1.54 pg reported by Marie and Brown (1993). The observations demonstrated that genome size in tetraploid plants had been successfully doubled to 3.74 pg confirming the effectiveness of colchicine treatment (**Figure 4**).

**Figure 5:**
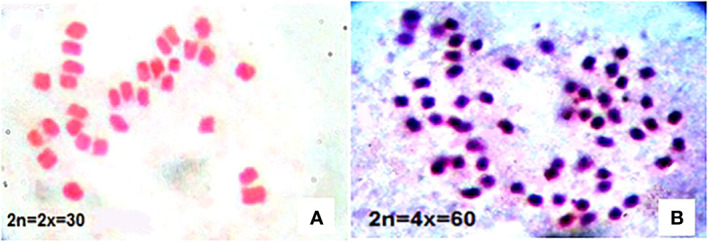
Chromosome number in diploid **(A)** and induced tetraploid **(B)** thyme.

To ensure the ploidy level of plants, the number of chromosomes was counted in root tip cells of candidate tetraploid plants confirmed by flow cytometry. The number of chromosomes in diploid thyme was 2n = 2x = 30. Although some plants had 57 or, 58 chromosomes, plants with exactly 60 chromosomes were chosen as autotetraploid (**Figure 5**).

### Morphological traits

There was a growth stunt in tetraploid plants up to two months after treatment. Tetraploid plants exhibited taller and thicker stems, dense branching, and larger leaves (**Table 1**).

**Table 1:** Morphological traits in diploid vs. tetraploid thyme.

Ploidy	Plant height (cm)	Internode distance (mm)	Stem diameter (mm)	Leaf thickness (mm)	Leaf length (mm)	Leaf width (mm)
2X	22.90	20.16	0.78	0.28	6.29	3.40
4X	26.50 *	15.84**	1.12**	0.4**	7.27 *	4.44**

*,** significantly different at 5 and 1%, respectively.

### Anatomical characteristics

The results showed that tetraploid plants had lower number but larger stomata than diploid plants. Trichome size was also significantly increased (**Table 2**). The number of chloroplasts and mitochondria in tetraploid plants increased by 1.66 and 1.63 times, respectively (**Table 2**) while there was no significant difference in their size compared to control diploid ones.

**Table 2:** Mean of anatomical traits in diploid and tetraploid thyme.

Ploidy	Stomata density	Stomata length (micm)	Stomata width (micm)	Guard length (micm)	Trichome size (micm)	Chloroplast number	Mitochondria number
2x	22	0.91	0.45	1.47	7.81	12.5	60.7
4x	13	1.38^*^	0.40 ^ns^	2^*^	10^**^	20.7^*^	99.1^*^

*,** and ns significantly different at 5, 1%, and non-significant, respectively.

### Expression of cytochrome genes

**Figure 6:**
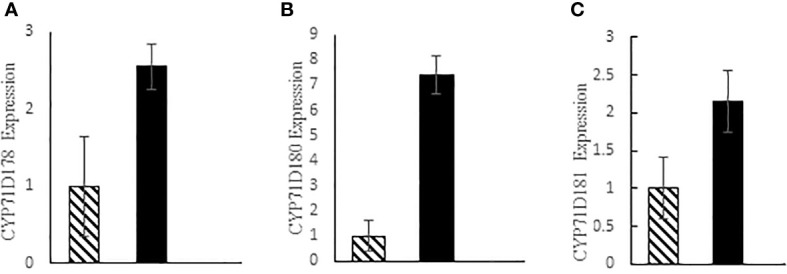
Expression level of three cytochrome genes; **(A)** CYP71D178, **(B)** CYP71D180 and **(C)** CYP71D81 in thyme; diploid (hatched bars) and tetraploid (black bars).

Real-time PCR of Cytochrome genes involved in thymol and carvacrol biosynthesis indicated that the expression of CYP71D178, CYP71D180 and CYP71D181 genes increased by 3.27-fold, 7.39-fold, and 2.15-fold in tetraploid plants compared to diploid controls, respectively (**Figure 6**).

### Biochemical traits

Content of main compounds in tetraploid and diploid plants are shown in **Table 3**. Essential oil content, thymol, carvacrol, γ-terpinene, and linalool increased in tetraploid plants, while p-Cymene, Camphor and chlorophyll content were decreased.

**Table 3:** Main compounds in tetraploid and diploid Thyme.

Compound	Diploid (%)	Tetraploid (%)	Change%
Essential Oil	0.85	1.4	64.7
Thymol	26.9	37.9	40.9
Carvacrol	11.8	14	18.6
γ-Terpinene	14	18.1	29.3
p-Cymene	12.1	9	-25.6
Linalool	1.7	2.9	70.6
Camphor	2.5	1.4	-44

## Discussion

### Polyploidy induction, cytometry and cytogenetic tests

Induced polyploidy has been used by scientists in several field, medicinal and ornamental crops in order to enlarge the plant organs or to enhance secondary metabolites production (Chen et al., 1945; Dhawan and Lavania, 1996; Zeng et al., 2006; Sattler et al., 2016; Salma et al., 2017; Madani et al., 2021). The probable decline in seed setting of thyme induced polyploids would not make any barrier in its cultivation, as the plant can be asexually propagated through cuttings.

In this research, we made an attempt to double the number of chromosomes in *Thymus vulgaris* in an *in vivo* condition. The results indicated that applying 0.3% colchicine on apical meristem of four-leaf stage seedlings for 6 hours was optimum treatment for polyploidy induction leading to maximum survival and the number of tetraploid plants (**Figures 2**, **3**). Tavan et al. (2015) also found 0.3% colchicine (although for 12 h) as the most efficient condition for inducing polyploidy in *Thymus persicus* nodal shoot tip explants. Flow cytometry output (**Figure 4**) demonstrated doubled genome size in tetraploid plants (3.74 pg) and cytogenetic analysis confirmed the increase of chromosome number from 2n=2x=30 in diploids to 2n=4x=60 in induced tetraploids (**Figure 5**).

Previous studies on *T. vulgaris* have reported tetraploid induction and its effect on essential oil production, but those procedures involved nodal segments and oryzalin, instead of colchicine (Shmeit et al., 2020; Navrátilová et al., 2021).

One of the concerns of *in vitro* ploidy induction is the low number of polyploid plants finally obtained, as too many plantlets are lost during tissue culture procedure from explants until mature plants. For example, polyploidy rate has been reported 7.5%, 2.3-10% and 7.8% in Shmeit et al. (2020); Navrátilová et al. (2021), and Tavan et al. (2015) experiments, respectively, while it was 24.8% in our study (**Figure 3**). *In vivo* polyploidization does not need any well-equipped tissue culture lab and this might be another privilege.

### Morphological changes in tetraploids

There was a growth stunt in tetraploid plants up to two months after treatment probably due to toxic damage and degradation caused by colchicine (Swanson, 1957). Chen and Gao (2007) also found a growth retardation in *Astragalus membranaceus* in early weeks and later normal growth following ploidy induction.

Tetraploid plants exhibited taller and thicker stems, dense branching, and larger leaves (**Table 1**), likely as a result of increased cell size (Comai, 2005). This result is in close agreement with previous studies. Shmeit et al. (2020) reported increased plant weight, height, leaf length, breadth and thickness and Navrátilová et al. (2021) demonstrated shorter internodes, darker and rounded leaves, and more compact appearance in induced tetraploid plants of thyme.

A correlation between ploidy level and morphological characteristics has been reported in numerous plant species such as *Phlox subulata* (Zhang et al., 2008), *Tanacetum parthenium* (Majdi et al., 2010), *Gerbera jamesonii* (Gantait et al., 2011), *Lychnis senno* (Nonaka et al., 2011), *Ullucus tuberosus* (Viehmannova et al., 2011), and *Ocimum basilicum* and *Dracocephalum moldavica* (Omidbaigi et al., 2010; Omidbaigi et al., 2012). Larger leaves in induced tetraploids has been reported in *Platanus acerifolia* (Liu et al., 2007), *Tanacetum parthenium* (Majdi et al., 2010), *Dracocephalum moldavica* (Omidbaigi et al., 2010). Lower height, shorter roots, thicker stems and darker leaves have been observed in *Thymus persicus* induced tetraploids by Tavan et al. (2015). Sabzehzari et al. (2019) also concluded that induced tetraploid plants of Ispaghul (*Plantago psyllium*) were larger than their intact diploids in terms of height, leaf thickness and seed size.

### Anatomical changes in tetraploids

It has been observed by several researches that tetraploid induction may significantly alter the length, width and number of stomata (Cohen and Yao, 1996; Beck et al., 2003; Yang et al., 2006; Liu et al., 2007). Tetraploid plants exhibited a reduced number of stomata compared to diploid plants, but the individual stomata were larger in size (**Table 2**). Longer and wider stomata and reduced stomatal density were reported by Tavan et al. (2015) in induced tetraploids of *Thymus persicus*. Javadian et al. (2017) detected increased size of leaves and stomatal cells, while decreased stomata density in *Linum album* tetraploids. Studies indicate that in line with the increase in ploidy level, the cell incorporates more chromosomes into the nucleus, leading to an increase in cell size (Byrne et al., 1981; Kondorosi et al., 2000; Comai, 2005; Ng et al., 2011).

Our findings revealed that the number of chloroplasts and mitochondria increased by 1.66 and 1.63 times in tetraploid plants, respectively (**Table 2**) without any alteration in their size compared to control diploid ones. Increased chloroplast number in polyploid plants have been reported in tomato (Koornneef et al., 1989), *Triticum monococum* (Hawke and Leech, 1990), watermelon (Sari et al., 1999), *Medicago* spp (Ghanavati et al., 2004), and *Onobrychis* spp (Ghanavati and Eskandari (2011). A positive correlation between the mean number of chloroplasts and the number of chromosomes was discovered by Frandsen (1968) in potato. Ordoez et al. (2017); Alsahlany et al. (2019) and Kramer and Bamberg (2019) recently recommend chloroplast counting as an efficient and inexpensive method for breeders to easily differentiate ploidy between diploid and tetraploid potato. Increased number of chloroplasts (and mitochondria) in tetraploids could be arisen form increase in the copy number of genes controlling their biogenesis in the nucleus (Leech et al., 1981; Ghanavati et al., 2004).

### Expression of cytochrome genes

Evidence suggest that polyploidy activates some mechanisms in cell effecting DNA template and also transcription and translation leading to decrease, increase or even silence the gene expression so that it can influence physiological and morphological traits of the plant (Adams and Wendel, 2005). In line with our findings, an enhance in transcription level of benzylisoquinoline biosynthesis genes in *Papaver bracteatum* tetraploid plants has been observed by Madani et al. (2019). Braynen et al. (2021) reported the up-regulation of floral transition genes in autotetraploid *Brassica rapa*, too.

The expression of CYP71D178, CYP71D180 and CYP71D181 genes appeared to be much higher in tetraploid plants compared to diploid controls in our study (**Figure 6**). This result justifies the higher amount of thymol and carvacrol in tetraploid plants (**Table 3**). CYP71D178, CYP71D179 and CYP71D82 are proposed to be thymol synthases while CYP71D180 and CYP71D181 being carvacrol synthases Crocoll et al. (2011).

Cytochrome genes are known to express enzymes catalyzing a number of oxidations of terpene metabolism and are likely to be involved in the reactions from γ-terpinene to thymol and carvacrol. Crocoll et al. (2010) isolated eleven cytochrome P450 gene sequences from oregano, thyme and marjoram and suggested a pathway for thymol and carvacrol starting with the formation of γ-terpinene by a monoterpene synthase. The second step appeared to be catalyzed by cytochrome P450s in a two-step oxidation. Krause et al. (2021) elucidated biosynthetic pathway of thyme main compounds suggesting that the aromatic backbone of thymol and carvacrol is formed by P450 mono-oxygenases in combination with a dehydrogenase via an unstable intermediate. Two additional P450s of the CYP76S and CYP736A subfamilies catalyze the hydroxylation of thymol and carvacrol to thymohydroquinone.

### Biochemical changes in tetraploids

Essential oil content in tetraploid plants was 64.7% higher than that in diploid plants as the content increased from 0.85 to 1.4 (**Table 3**). These results are almost the same as findings of Shmeit et al. (2020) who reported 0.81% and 1.19% essential oil in diploid and tetraploid thyme plants, respectively. Thymol, carvacrol and γ-terpinene, as main medicinal compounds of thyme (Galovičová et al., 2021), were found to increase by 40.9%, 18.6% and 29.3%, respectively, in tetraploid plants (**Table 3**). The content of thymol and carvacrol increased by 18.01% and 0.49%, respectively in tetraploid thyme plants developed by Shmeit et al. (2020). Navrátilová et al. (2021), however, observed no difference in total essential oil between diploids and induced tetraploids, although single analyte concentrations *e.g.*, thymol and carvacrol were increased. Tohidi et al. (2021) found that carvacrol in natural tetraploid populations of thyme was higher than diploid ones.

Evidence suggests that γ-terpinene is primary substrate for to thymol and carvacrol synthesis (Crocoll et al., 2010; Lima et al., 2013). So, its rise in tetraploids seems to have increased thymol and carvacrol content as shown in **Table 3**. An increase of 35.6% and 24% in essential oil have been reported in *Carum carvi* (Dijkstra and Speckmann, 1980) and *Dracocephalum moldavica* (Omidbaigi et al., 2010) in induced tetraploids, respectively. A 50% rise in artemisinin content in *Artemisa annua* (Lin et al., 2011), 5.8-times higher thebaine in *Centella asiatica* (Madani et al., 2019), and 52–152% increase in alkaloid production in *Datura stramonium* (Berkov and Philipov, 2002) have been documented in the literature, too.

Chlorophyll content was decreased from 2.01 to 1.6 mgg^-1^ fresh weight (20.4%). Decline in chlorophyll content justifies the darker color in tetraploid plants leave as reported in *Urgenia indica* (Phulari, 2011), lemon (Afshar Mohammadian et al., 2012), *Thymus persicus* (Tavan et al., 2015) and *Thymus vulgaris* (Navrátilová et al., 2021).

## Conclusion

In this research the number of chromosomes of *Thymus vulgaris* was doubled from 30 to 60 by an *in vivo* protocol and its impact on morphology, anatomy, phytochemistry and gene expression was studied. Application of 0.3% colchicine for 6 hours at 4-leaf seedling appeared to be optimum treatment for polyploid induction leading to maximum survival and the number of tetraploid plants Number of chloroplasts and mitochondria, the number of stomata cells and guard cells length increased. Tetraploid plants outperformed the diploid plants by 64.7% in essential oil, 40.9% in thymol and 18.6% in carvacrol content. The findings of this research suggest that induced polyploidy has the potential to enhance the production of secondary metabolites in thyme, thus providing opportunities for its genetic improvement.

## Data availability statement

The raw data supporting the conclusions of this article will be made available by the authors, without undue reservation.

## Author contributions

VM has proposed the idea and was project leader and the main author. ST, MA, and HM conducted the experiment under the supervision of VM. All authors contributed to the article and approved the submitted version.
